# Retinoic acid-loaded polymeric nanoparticles induce neuroprotection in a mouse model for Parkinson's disease

**DOI:** 10.3389/fnagi.2015.00020

**Published:** 2015-03-06

**Authors:** Marta Esteves, Ana C. Cristóvão, Tatiana Saraiva, Sandra M. Rocha, Graça Baltazar, Lino Ferreira, Liliana Bernardino

**Affiliations:** ^1^Faculty of Health Sciences, Health Sciences Research Centre, University of Beira InteriorCovilhã, Portugal; ^2^Center for Neuroscience and Cell Biology, University of CoimbraCoimbra, Portugal; ^3^Biocant – Center of Innovation in BiotechnologyCantanhede, Portugal

**Keywords:** retinoic acid, nanoparticles, neuroprotection, dopaminergic neurons, Parkinson's disease

## Abstract

Retinoic acid (RA) plays an important role in the commitment, maturation and survival of neural cells. Recently, RA was pointed as a therapeutic option for some neurodegenerative diseases, including Parkinson's disease (PD). The administration of RA has been defying, and in this sense we have previously developed novel RA-loaded polymeric nanoparticles (RA-NPs) that ensure the efficient intracellular transport and controlled release of RA. Herein, we show that nanoformulation as an efficient neuroprotective effect on dopaminergic (DA) neurons in the 1-methyl-4-phenyl-1, 2, 3, 6-tetrahydropyridine (MPTP) induced mouse model for PD. The results showed that the RA-NPs administration induced a significant reduction of DA neuron loss in the substantia nigra (SN) as well as their neuronal fiber/axonal innervations in the striatum. Furthermore, we observed an increase in the expression levels of the transcription factors Pitx3 and Nurr1 induced by RA-NPs, showing its supportive effect on the development and functional maintenance of DA neurons in PD. This is the first study showing that RA-NPs can be an innovative strategy to halt the progression of PD pathogenesis, suggesting that this nanoformulation could be of particular interest for the development of new approaches for PD therapeutics.

## Introduction

Retinoic acid (RA) is a metabolic product of vitamin A (retinol) which plays important roles in the development of mammalian nervous system (Maden, 2007). RA has been also highlighted as a therapeutic option for some neurodegenerative disorders, including in Parkinson's disease (PD). RA is likely to be important for midbrain dopaminergic (mDA) neurons since its receptors and RA-synthesizing enzymes are highly expressed both in these neurons and their target regions (McCaffery and Drager, 1994). The RA signal is transduced by specific nuclear receptors: retinoic acid receptors (RAR) and retinoid X receptors (RXR), which are members of the nuclear receptor superfamily (Kastner et al., 1997). These receptors heterodimerize and bind to a DNA sequence called retinoic acid-response element (RARE) and induce gene transcription (Bastien and Rochette-Egly, 2004). Several evidences suggest an involvement of RA signaling in the development, maintenance and protection of the nigrostriatal pathway; however the cellular and molecular mechanisms underlying these effects are not yet known. Most importantly, it was shown that stimulation of RAR with RAR agonist AM80, prevented DA cell loss induced by lipopolysaccharide (LPS) in the substantia nigra (SN) (Katsuki et al., 2009). In accordance, it was shown by Yin and collaborators that the intranasal delivery of RA reduces neurodegeneration of DA neurons induced by 6-hydroxidopamine (6-OHDA) (Yin et al., 2012). Clinically, there is a marked reduction in the expression levels of the enzyme retinaldehyde dehydrogenase 1 (RALDH1), which is necessary for the conversion of retinaldehyde to RA, in the mDA neurons of post-mortem brain PD patients (Galter et al., 2003). The RALDH1 expression levels found in peripheral blood have been recently reported as a candidate biomarker for PD diagnosis (Grunblatt et al., 2010). However, it is not possible to know if this decrease in RALDH1 gene expression precedes the onset of PD or is a consequence of the degenerative process. It may be possible that the reduced availability of RA in the midbrain might be due to the reduced RALDH1 expression, which in turn increases the susceptibility of the mDA neurons to the degenerative processes, pushing the balance toward neuronal death instead of neuroprotection. Mutations in the gene coding for this enzyme was also shown to represent a genetic risk factor for human PD either alone or in conjunction with other environmental risk factors (Buervenich et al., 2005).

The administration of RA is challenging due to its undesirable properties, like poor water solubility, short half-life, requires a fine-tuning of the concentration window to achieve its effects, posing difficulties in the delivery of therapeutic doses (Szuts and Harosi, 1991). Therefore, we recently developed nanoparticles (NPs) loaded with all-trans retinoic acid (atRA) as an alternative to control the undesired side effects and to ensure intracellular transport and controlled release of RA. A successful approach was achieved by approximately 200 nm-sized NPs which were rapidly taken up by cells, delivering RA intracellularly (Maia et al., 2011). Several NPs formulations have been reported for the controlled release of this molecule (Castro et al., 2007; Narvekar et al., 2012); however, none of these formulations were designed to deliver RA for DA neuroprotection. It was shown previously by our group that the internalization of the atRA-loaded polymeric NPs (RA-NPs) has a minimal effect on cell viability and proliferation but enhanced neurogenesis at the subventricular zone stem cell niche, both *in vitro* and *in vivo* (Maia et al., 2011; Santos et al., 2012).

In the present study, we examined the putative neuroprotective effect of NPs-encapsulated RA in a PD mouse model. In addition to that, the expression of Nurr1 and Pitx3 both at mRNA and protein levels were examined in SN and striatum as these two transcription factors are involved in the development, survival and specification of DA neurons. With the present results we have proved that this RA-NPs formulation may create a favorable environment to protect DA neurons in the nigrostriatal pathway, as well as by preventing the decrease of mRNA and protein expression of transcription factors involved in DA neurons maintenance. This work reports for the first time a RA-releasing nanoformulation as an efficient strategy to prevent the onset of PD, and possibly to open new therapeutic perspectives for the treatment of other neurodegenerative diseases.

## Materials and methods

### Animals

Young adult (2–3 months old) and old (25–26 months old) male C57BL6 mice were used for this study. All animals were handled in accordance with protocols approved by the national ethical requirements for animal research, and in accordance with the Directive 2010/63/EU of the European Parliament and the Council on the protection of animals used for scientific purposes. Mice were kept in appropriate cages, under temperature-controlled conditions with a fixed 12 h light/dark cycle, food and water freely available. All efforts were made to reduce the number of animals to be used for the study and to minimize their suffering.

### Stereotaxic injection

Both young adult and old mice were anesthetized with intraperitoneal (i.p.) injection of ketamine (90 mg/kg of mouse weight) and xylazine (10 mg/kg of mouse weight) and placed in a stereotaxic frame. The skull was exposed and the scales were defined after setting the zero at the bregma point. Mice were then unilaterally injected in the right lateral striatum (X,AP: +0.6; Y,ML: −1.8; Z,DV: −2.8 mm, Paxinos and Franklin, 2001), which was considered the ipsilateral side, with 100 ng/ml RA-NPs (dissolved in sterile phosphate buffer saline, PBS: NaCl 140 mM, KCl 2.7 mM, KH_2_PO_4_ 1.5 mM and Na_2_HPO_4_ 8.1 mM, pH 7.4), 100 ng/ml blank NPs (void formulation; dissolved in PBS), 4 nM or 10 μM solubilized atRA (dissolved in dimethyl sulfoxide (DMSO); final dilution of 1:250,00000 and 1:10,000, respectively), or sterile 0.1 M PBS (vehicle) through a 10 μl Hamilton syringe at a speed of 0.2 μl/min over 5 min. The contralateral side was the left lateral striatum and remains uninjected. The atRA was used as this is the prevalent functionally active isoform of RA. RA-NPs, blank NPs and solubilized atRA were prepared freshly just before the injections and the solubilized atRA solution was protected from light and kept on ice until injection. The concentrations of RA-NPs, blank NPs and solubilized atRA were chosen based in previous studies developed by us (Maia et al., 2011; Santos et al., 2012). The vehicle used for the NPs formulations was 0.1 M sterile PBS.

### MPTP-induced lesion and tissue processing

**Figure 1:**
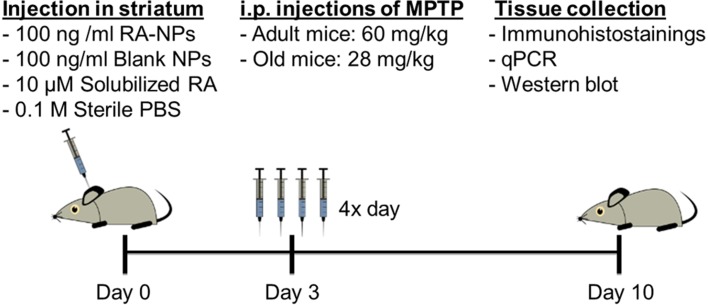
**Timeline for experimental treatments and assays performed *in vivo***.

MPTP administration was given 3 days after intrastriatal injections of nanoparticles formulations. MPTP (Sigma-Aldrich, St. Louis, MO, USA) was dissolved in sterile 0.9% NaCl and injected via i.p. in four sessions separated by 2 h intervals. The dose used was 15 mg/kg body weigh in adult mice (Kong et al., 2008) and 7 mg/kg body weight in old mice (Peng and Andersen, 2011), been the total dose after the 4 injections of 60 and 28 mg/kg, respectively. The saline group animals, subjected to the same stereotaxic procedure, received an equivalent volume of sterile 0.9% NaCl. Seven days after the MPTP exposure (Figure 1), one group of animals was anesthetized with ketamine and euthanized by transcardial perfusion with 0.9% NaCl followed by perfusion with 4% paraformaldehyde (PFA) and the brains were then recovered. Brains were post-fixed by immersion in 4% PFA solution, transferred to 30% sucrose (in PBS) and then frozen. Afterward, brains were coronally sectioned at a thickness of 35 μm from the frontal pole to the midbrain on a freezing cryostat-microtome (Leica CM 3050S, Leica Microsystems, Nussloch, Germany) at −20°C. The sections encompassing the SN and the striatum of each animal were collected sequentially to six well of 24-well plate, and stored in PBS supplemented with 0.02% sodium azide at 4°C, until processed for immunohistostainings. Another group of animals was euthanized by spinal cord dislocation, the brains were removed and the regions of interest, SN and striatum, were quickly dissected from both hemispheres and stored at −80°C for RNA and protein expression analysis by quantitative real-time polymerase chain reaction (qPCR) and western-blot, respectively.

### Tyrosine hydroxylase (TH) immunohistostaining

The TH immunohistostaining was carried out to detect DA neurons in the SN and striatal TH fibers in the striatum. Coronal sections were incubated on a 10 mM citrate solution (pH 6.0) at 80°C for 30 min for antigen retrieval. After cooled to room temperature (RT), sections were placed in water for 5 min and then washed with PBS-Tween 20 (PBS-T). Sections were then incubated with PBS containing 10% fetal bovine serum (FBS) and 0.1% Triton X-100 for at least 1 h at RT. Endogenous peroxidase activity was inhibited by incubation with 3% hydrogen peroxide (H_2_O_2_) in water for 10 min at RT and the sections were then washed with PBS-T. Sections were incubated with primary antibody mouse anti-TH (dilution 1:1000; Transduction Laboratories, Lexington, NY, USA) diluted in PBS containing 5% FBS, overnight at 4°C. After several rinses with PBS-T, sections were incubated with a biotinylated goat anti-mouse secondary antibody (dilution 1:200; Vector Laboratories, Burlingame, CA, USA) diluted in PBS containing 1% FBS for 1 h at RT. The avidin-biotin peroxidase complex reagent (Vectastain ABC KIT, Vector Laboratories Inc., Burlingame, CA, USA) was then added for at least 30 min at RT. The reaction product was visualized using 3, 3′-diaminobenzidine (DAB) (Sigma) in Tris buffer saline (TBS: 20 mM Tris and 137 mM NaCl solution, pH 7.6) containing 0.08% H_2_O_2_ until color develops (5–10 min). The reaction was stopped by adding TBS. Sections were mounted on slides, dried and dehydrated in graded ethanol dilutions, cleared in xylene and cover slipped using a permanent mounting medium (Entellan, Merck, NJ, USA). Quantitative analysis of DA neurons in SN was carried out by serial section analysis of the total number of TH positive (TH+) neurons throughout the rostro-caudal axis. Only the region corresponding to the SN was carefully delineated, according to the mouse brain atlas of Paxinos and Franklin (Paxinos and Franklin, 2001), and the total number of TH+ neurons in the full extent of this structure was counted blind to control, per section in each hemisphere. The total number of TH+ neurons for each representative mesencephalic section was quantified in four coronal sections per mouse serially selected from −2.80 to −3.80 mm relative to bregma, under the magnification of 10x at the Zeiss Axiovert 200 imaging microscope (Axiobserver Z1, Carl Zeiss, Oberkochen, Germany). Quantitative analysis of the intensity and area occupied by TH+ fibers staining in striatum was carried out in four serially selected coronal sections of striatum, from 1.10 to 0.38 mm relative to bregma of each mouse, selected throughout the rostro-caudal axis, under the magnification of 5x at the Zeiss Axiovert 200 imaging microscope.

### Total RNA extraction and cDNA synthesis

Total RNA was extracted from the striatum and SN using illustra RNAspin Mini RNA isolation Kit (GE Healthcare UK Limited, Buckinghamshire, UK, Cat no. 25-0500-71) according to manufacturer's protocol (Santos et al., 2012). Briefly, the samples were first lysed in lysis solution containing guanidine thiocyanate which ensured the inactivation of RNases. Samples were applied to spin mini filters to filtrate the lysate and the remaining filter was discarded. Afterwards, 70% ethanol was added to the filtrate to complex and precipitate nucleic acids. Samples were then transferred to spin mini columns where total RNA bound to the membrane. Then, salts were removed from silica membrane by the addition of desalting buffer therefore allowing a more efficient DNA digestion with DNase I. After incubation with DNase I, the column was washed and dried by the addition of a series of wash buffers promoting inactivation of DNase I and removing contaminants from the membrane-bound RNA, allowing the purification of high-quality mRNA enriched solution. At the end, mRNA samples were dissolved in 25 μl of RNase-free water and the total amount of mRNA was quantified by the nanophotometer (Implen) at 260 nm, and the purity was determined by measuring the 260/280 nm ratio. cDNA synthesis was performed using transcriptor first strand cDNA synthesis Kit (Roche, Basel, Switzerland, Cat no. 4379012001) according to the manufacturer's instructions. Briefly, total mRNA extracted from adult (0.2 μg) and old mice (0.4 μg) tissue samples was mixed with 1 μl anchored-oligo (dT) 18 primers, 4 μl reverse transcriptase reaction buffer 5x, 0.5 μl RNase inhibitor, 2 μl deoxynucleotide mix (dNTPs), 0.5 μl reverse transcriptase and sterile water in a 20 μl final volume. The reaction was performed at 55°C for 30 min and stopped at 85°C for 5 min step by a thermocycler (Biometra, Goetting, Germany). The samples were then stored at −80°C until further use.

### Quantitative real-time PCR (qPCR)

The qPCR assays for gene expression analysis of Nurr1 in the striatum and SN and Pitx3 in the SN were performed by adding 2 μl of cDNA sample, 10 μl SYBR Green Supermix (BioRad Laboratories, CA, USA), 1/10 dilution of each primer (according to primers datasheet) and RNase free water to a 20 μl total volume. The reaction was initiated with activation of Taq polymerase by heating at 94°C during 3 min followed by 40 cycles of a 15 s denaturation step at 94°C and a 30 s annealing and elongation step at 60°C. Validated primer sets (GAPDH, Nurr1 and Pitx3) were obtained from selected QuantiTect Primer Assays (Qiagen, Austin, Texas). The fluorescence was measured after the extension step by the iQ5 Multicolor Real-time PCR detection system (BioRad). After the thermocycling reaction, a melting curve was performed with slow heating, starting at 55°C and with a rate of 0.5°C per 10 s, up to 95°C. The assay included a non-template control (sample was substituted by RNase- Dnase-free sterile water). All reactions ran in duplicates. The threshold cycle (Ct) was measured in the exponential phase and therefore was not affected by the possible limiting components in the reaction. Data analysis was performed with BioRad iQ5 software (BioRad). Fluorescent reading from qPCR was quantitatively analyzed by determining the difference of Ct (ΔCt) between Ct of the target gene and GAPDH control housekeeping gene using the comparative Ct method as described by Pfaffl's formula (Pfaffl, 2001).

### Protein extraction and western-blot

For the western blotting analysis, after dissecting the SN and striatum from all brain tissues, these regions were lysed on ice in RIPA buffer (50 mM Tris/HCl, pH 8.0, 150 mM NaCl, 2 mM sodium orthovanadate, 1% Nonidet-P40, 0.5% sodium deoxycholate, 0.1% SDS, and 1% of a protease inhibitor mixture containing AEBSF, pepstatinA, E-64, bestatin, leupeptin, and aprotinin). The soluble fraction was obtained and equal amounts of total cell lysates were loaded in each lane of a 10% polyacrylamide gel. After electrophoresis and transfer onto a polyvinylidene difluoride (PVDF) membrane, specific protein bands were detected using appropriate primary antibodies (rabbit anti-Pitx3; rabbit anti-Nurr1; mouse anti-TH and mouse anti-GAPDH and respective secondary antibodies conjugated to horseradish peroxidase (anti-rabbit or anti-mouse) followed by Enhanced Chemiluminescence (ECL) detection. Densitometric analyses were performed by using the ImageQuant software (Bio-Rad).

### Statistical analysis

**Figure 2:**
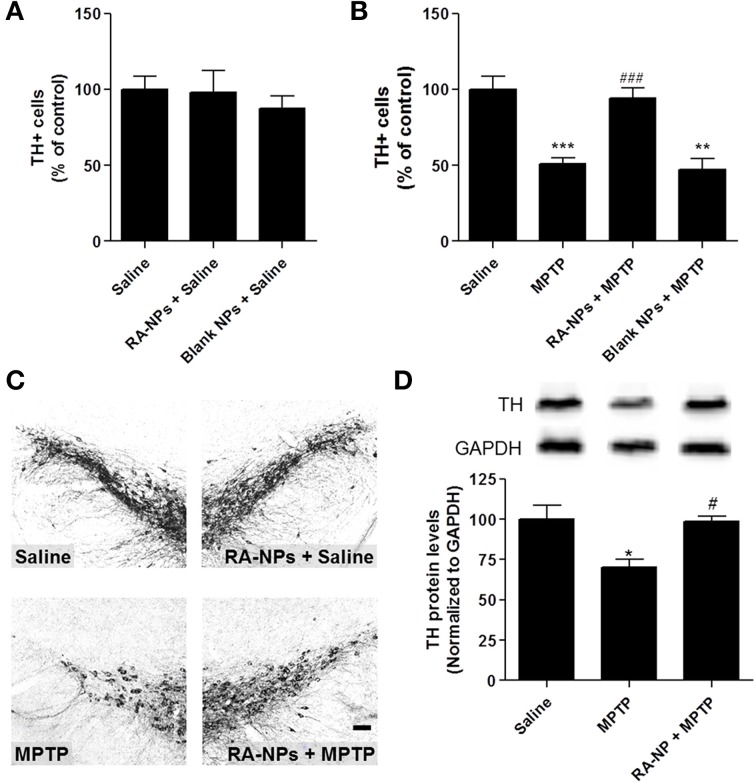
**RA-NPs promote the survival of TH+ cells in the MPTP-injured SN**. Adult mice received RA-NPs, blank NPs or solubilized RA by stereotaxic injections in the right striatum. MPTP or saline (0.9% sterile NaCl) were administered intraperitoneally (i.p.) 3 days after stereotaxic injections of NPs formulations. The SN region was collected 7 days after MPTP or saline i.p. injections. **(A)** Quantitative analysis of TH+ cells in the SN of control mice (saline, i.p.) injected with RA-NPs (RA-NPs + saline) or blank NPs (blank NPs + saline). **(B)** Quantitative analysis of TH+ cells in the contralateral SN of MPTP-treated mice and in the ipsilateral SN of RA-NPs- and blank-treated MPTP mice. **(C)** Representative photomicrographs of SN sections immunostained for TH of mice treated with RA-NPs followed by the i.p. injection of saline or MPTP. Scale bar: 50 μm. **(D)** TH protein levels in the SN of saline, MPTP or RA-NPs+ MPTP treated mice. A representative Western blot for 60 kDa TH and 37 kDa GAPDH expression is shown. Data are expressed as the percentage of control and represent the mean ± SEM (*n* = 3–8 mice). ^***^P < 0.001, ^**^P < 0.01 and ^*^P < 0.05 compared to saline, ^###^*P* < 0.001 and ^#^*P* < 0.05 compared to MPTP, using One-Way ANOVA followed by Dunnett's test.

**Figure 3:**
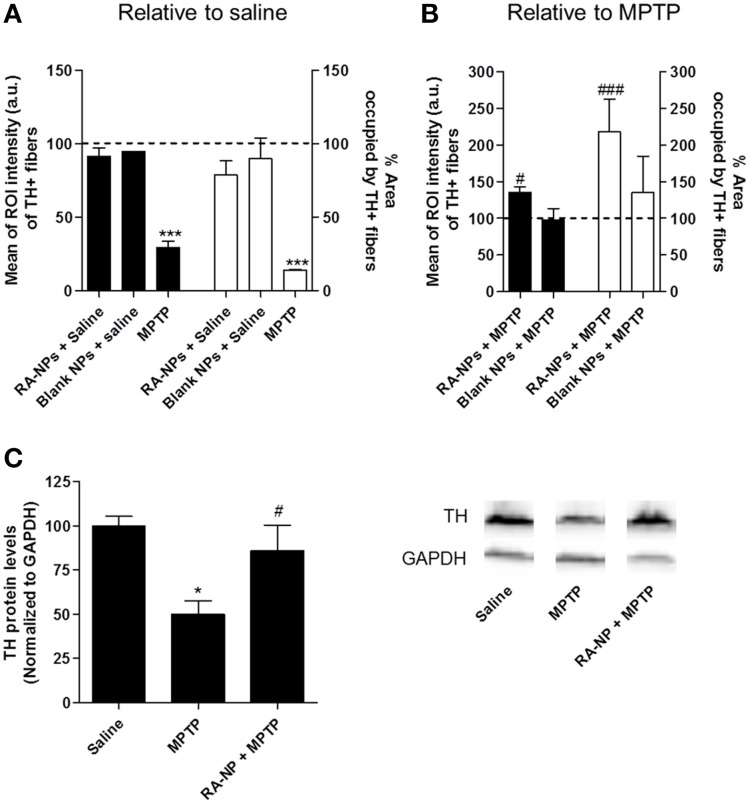
**RA-NPs increase TH+ fibers immunoreactivity in the MPTP-injured striatum**. Adult mice received RA-NPs or blank NPs by stereotaxic injections in the right striatum. MPTP or saline were administered i.p. 3 days after stereotaxic injections of NPs formulations. The striatum region was collected 7 days after MPTP or saline i.p. injections. **(A)** Quantitative analysis of the intensity and area occupied by TH+ fibers in control (contralateral side; set to 100%) and in RA-NPs- or blank-treated saline mice (ipsilateral sides). **(B)** Quantitative analysis of the intensity and area occupied by TH+ fibers in contralateral striatum of MPTP-treated mice (set to 100%) and in RA-NPs- and blank-treated MPTP mice (ipsilateral sides). Black bars: Intensity; White bars: Area. Data are expressed as the percentage of control **(A)** or MPTP **(B)** (both contralateral sides) and represent the mean ± SEM (*n* = 2–4 mice). ^***^P < 0.001 compared to saline and ^#^*P* < 0.05 and ^###^*P* < 0.001 compared to MPTP, using Two-Way ANOVA. **(C)** TH protein levels in the striatum of saline, MPTP or RA-NPs+ MPTP treated mice. A representative Western blot for 60 kDa TH and 37 kDa GAPDH expression is shown. Data are expressed as the percentage of saline and represent the mean ± SEM (*n* = 3 mice). ^*^P < 0.05 compared to saline and ^#^*P* < 0.05 compared to MPTP, using One-Way ANOVA followed by Dunnett's test.

**Figure 4:**
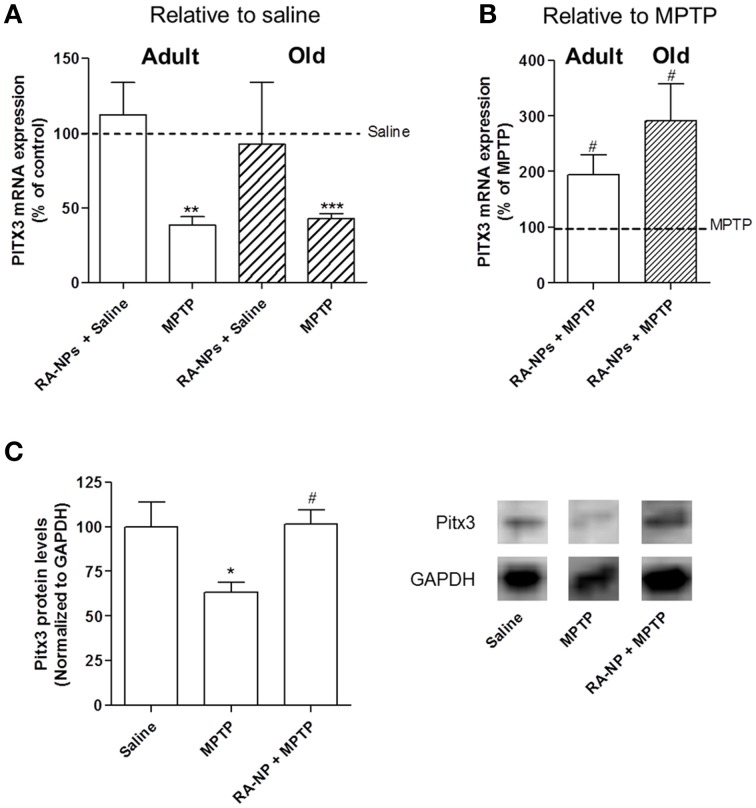
**RA-NPs induce the expression of transcription factor Pitx3 in the SN of adult and old mice exposed to MPTP**. Adult and old mice received RA-NPs by stereotaxic injections. Saline or MPTP were i.p. administered 3 days after stereotaxic injections of nanoformulations in the right striatum (ipsilateral side). Seven days after saline or MPTP injections, the SN region was collected for measurement of Pitx3 mRNA expression by qPCR. The expression levels were normalized to the GAPDH housekeeping gene. **(A,B)** Bar graphs indicate the percentage of Pitx3 mRNA expression in adult and old mice, expressed as the percentage of control **(A)** or MPTP **(B)**. **(C)** Pitx3 protein levels in the SN of saline, MPTP or RA-NPs+ MPTP treated adult mice. A representative Western blot for 32 kDa Pitx3 and 37 kDa GAPDH expression is shown. Data represent the mean ± SEM (*n* = 3–6 mice). ^***^P < 0.001, ^**^P < 0.01, and ^*^P < 0.05 compared to saline and ^#^*P* < 0.05 compared to MPTP, using One-Way ANOVA followed by Dunnett's test.

**Figure 5:**
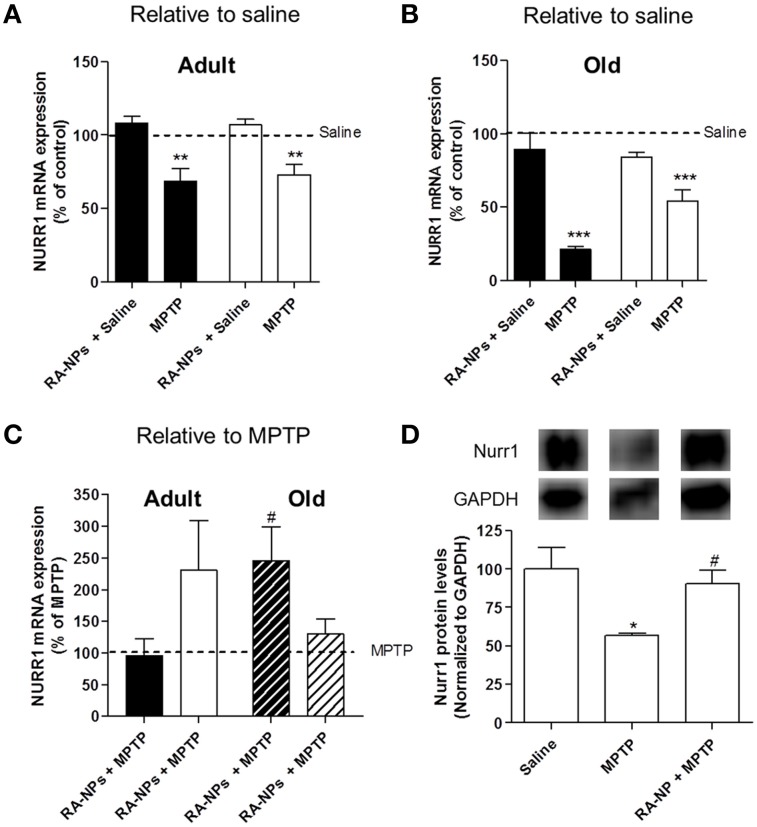
**RA-NPs induce the expression of transcription factor Nurr1 in MPTP-treated adult and old mice**. Adult and old mice received RA-NPs by stereotaxic injections. Saline or MPTP were i.p. administered 3 days after stereotaxic injections with nanoparticle formulations in the right striatum (ipsilateral side). Seven days after saline or MPTP injections, the SN and striatum regions were collected for measurement of Nurr1 mRNA expression by qPCR. The expression levels were normalized to the GAPDH housekeeping gene. **(A,B)** Bar graphs indicate the percentage of Nurr1 mRNA expression in adult and old mice, respectively, expressed as the percentage of control (saline, contralateral). **(C)** Bar graph indicate the percentage of Nurr1 mRNA expression in adult and old mice, relative to MPTP. Black bars: striatum; White bars: SN. **(D)** Nurr1 protein levels in the SN of saline, MPTP or RA-NPs+ MPTP treated adult mice. A representative Western blot for 66 kDa Nurr1 and 37 kDa GAPDH expression is shown. Data represent the mean ± SEM (*n* = 3–7 mice). ^***^P < 0.001, ^**^P < 0.01, and ^*^P < 0.05 compared to saline and ^#^*P* < 0.05 compared to MPTP using One-Way ANOVA followed by Dunnett's test.

Statistical analysis of group differences was performed using GraphPad Prism 5.0 (GraphPad Software Inc., San Diego, CA, USA) by one-way analysis of variance (ANOVA) followed by Dunnett's test for comparison with control condition (Figures 2, **4**, **5**) or Two-Way ANOVA (Figure 3). Control and MPTP values correspond to the contralateral sides of saline- and MPTP-treated mice, respectively. All other conditions correspond to ipsilateral sides of saline- or MPTP-treated mice. Contra and ipsilateral conditions are explained above in the Stereotaxic Injection Section. Blank NPs were used as negative control. The data are expressed as percentages of values obtained relative to the control or MPTP and are represented as the means ± standard error of mean (SEM). Statistical significance was considered relevant for *p* < 0.05.

## Results

### RA-NPs induce neuroprotection of the SN dopaminergic neurons against the MPTP-induced lesion

In this work we evaluated the putative neuroprotective effect of RA-NPs on a MPTP-induced mouse PD model. NPs were prepared by the electrostatic interaction of polyethylenimine (PEI, polycation) complexed with RA and dextran sulfate (DS, polyanion) according to a methodology reported previously by us (Maia et al., 2011). RA-NPs have a DS/PEI ratio of 0.2, yielding NPs that are approximately 220 nm in diameter and have a positive net charge (zeta potential of about 16 mV). RA-NPs developed by us ensure an efficient intracellular transport and controlled release of RA. We have also demonstrated that pH enabled the control of RA releasing from NPs showing that the RA exposed to pH 7.4 was rapidly released over 10 days followed by a steady increase until day 21 (Maia et al., 2011). This pH is roughly the pH found in the brain (Casey et al., 2010). In contrast to these days of exposure to RA when delivered in NP formulations, the half-life of free solubilized RA administered directly into the brain is only for few hours. First, we did not find any statistical difference in the percentage of TH+ cells in the SN of RA-NPs or blank NPs ipsilateral sides compared with the contralateral sides (control) of saline animals (Figure 2A). These results suggest that both RA- and blank-NPs did not interfere *per se* with the DA neuronal survival. The exposure to MPTP triggered approximately 50% reduction of TH+ cells in SN as compared with the saline animals (*p* < 0.001) (Figures 2B,C). The intrastriatal injection with RA-NPs significantly prevented the loss of DA neurons induced by MPTP (*p* < 0.001) (Figures 2B,C). Additionally, the levels of TH protein in the SN corroborate the above results, since the significant decrease of TH protein levels induced by MPTP exposure was reverted by the presence of RA-NPs (Figure 2D).

We also investigated the putative neuroprotective effect of 4 nM and 10 μM solubilized RA. The concentration of 4 nM corresponds to the payload of RA present in 100 ng/ml RA-NPs, whereas 10 μM is the concentration of solubilized RA capable of inducing a proneurogenic effect on SVZ cells *in vitro* (Maia et al., 2011). The neuroprotective effect triggered by RA released from NPs was more robust than the one observed with solubilized 4 nM or 10 μM RA (78.7 ± 3.9% of control, *n* = 2; 63.2 ± 4.7% of control, *p* < 0.05, *n* = 3; respectively). As expected, intrastriatal injections of blank NPs, used as a negative control, were not able to protect TH+ neurons against the MPTP toxicity, showing that RA is the source of neuroprotection for TH+ neurons. Altogether, these results suggest that the prophylactic administration of RA-NPs was very effective in protecting SN DA neurons against MPTP toxicity.

### RA-NPs support dopaminergic fiber striatal innervations against the MPTP-induced lesion

DA neuronal cell bodies present on the SN project axonal fibers toward the striatum. Therefore, we performed TH immunohistostainings in striatal coronal sections of adult mice in order to analyze the putative trophic effects driven by RA released from NPs on DA axonal fibers. The intensity and area occupied by TH+ immunoreactive fibers were quantified in saline- and MPTP-treated mice. No statistical difference was found in the intensity (black bars) and area (white bars) occupied by TH+ fibers in the contralateral striatum of saline-treated animals (line set to 100%) as compared to the ipsilateral side of the same animals injected with RA- or blank-NPs (Figure 3A). As expected, MPTP caused a significant decrease in the intensity and percentage of area occupied by TH+ fibers as compared to saline-treated animals (set to 100%) (Figure 3A). Pre-exposure to RA-NPs significantly protected the TH+ fibers against MPTP induced degeneration (Figure 3B). As expected, the blank NPs did not interfere with the MPTP toxicity (Figure 3B). In addition, the same tendency regarding the TH protein levels was also found in the striatum, reinforcing the neuroprotective effect of RA-NPs against MPTP DA toxicity. The significant decrease of TH protein levels induced by MPTP was prevented by the injection of RA-NPs (Figure 3C). These data suggest that RA-NPs might also support DA neuronal projections present in the striatum.

### RA-NPs modulate Nurr1 and Pitx3 mRNA and protein expression

To investigate possible molecular players involved in DA neuroprotection induced by RA-NPs, we then collected the SN and the striatum from adult (2–3 months) or old (25–26 months) mice. The aim of these experiments was to mimic the pathological environment occurring in PD, since the most predominant form of this disease (idiopathic) occurs in older individuals. We then analyzed the expression of transcription factors involved in DA development and survival—Nurr1 and Pitx3. Due to their regional-specific expression pattern (Smidt et al., 1997; Saijo et al., 2009), Nurr1 expression was evaluated in SN and striatum whereas Pitx3 expression was evaluated only in the SN.

As shown in Figure 4A, the intrastriatal injection with RA-NPs in saline-treated adult mice did not change the mRNA expression levels of Pitx3 as compared to the contralateral hemisphere (saline i.p.; line set to 100%). As expected, when mice were exposed to MPTP there was a significant decrease in the Pitx3 mRNA expression levels (*p* < 0.01). Accordingly, the decrease of Pitx3 mRNA levels is compatible with the loss of nearly 50% of TH+ neurons in SN induced by MPTP (Figure 2B). The administration of RA-NPs in the MPTP-treated adult mice significantly prevented the decrease of Pitx3 mRNA levels in the SN as compared to MPTP (*p* < 0.05) (Figure 4B). In accordance with previous data, blank NPs did not change Pitx3 mRNA expression as compared with MPTP-only exposed cells (106.6 ± 11% of MPTP; *n* = 3). The protein levels of Pitx3 were in accordance with the qPCR results, been significantly decreased in MPTP treated mice and not altered in the presence of RA-NPs, both compared with the saline group (Figure 4C).

qPCR analysis was also performed in old mice. First we showed that the striatal administration of RA-NPs did not significantly alter mRNA expression of Pitx3 as compared to saline-treated animals (Figure 4A). As expected, a robust decrease in Pitx3 mRNA levels was found in the hemisphere exposed to MPTP as compared to saline-treated animals (*p* < 0.001; Figure 4A). The administration of RA-NPs in MPTP-treated old mice significantly prevented the decrease of Pitx3 mRNA levels induced by MPTP (line set to 100%) (Figure 4B). Unfortunately we could not evaluate the TH protein levels in the SN of old mice due to the limited availability of mice with a substantially advanced age.

Nurr1 mRNA expression levels were also analyzed in the SN and striatum of both adult and old mice. The intrastriatal injection with RA-NPs in saline-treated adult mice did not change the mRNA expression levels of Nurr1 in the striatum and SN (saline mice; line set to 100%) (Figure 5A). When mice were exposed to MPTP there was a significant decrease in Nurr1 transcripts levels in both striatum and SN (*p* < 0.01) (Figure 5A). The administration of RA-NPs in MPTP-treated adult mice prevented the decrease of Nurr1 mRNA levels induced by MPTP (line set to 100%) (Figure 5C). In same conditions, no changes were detected in Nurr1 mRNA levels in the striatum exposed to both RA-NPs plus MPTP when compared with MPTP-only exposed contralateral striatum (line set to 100%) (Figure 5C). In accordance with previous data, blank NPs did not change Nurr1 mRNA expression in striatum and SN as compared with MPTP-only exposed cells (72 ± 14.4% and 85.4 ± 11.9% of MPTP, respectively; *n* = 3). Nurr1 protein levels in the SN of adult mice were also evaluated. RA-NPs striatal injection prevented the decrease of Nurr1 protein level as induced by MPTP exposure (Figure 5D).

qPCR analysis regarding the expression of Nurr1 mRNA was also performed in old mice. The striatal administration of RA-NPs in saline-treated mice did not significantly alter both SN and striatal mRNA expression of Nurr1 as compared to saline-treated animals (control; line set to 100%) (Figure 5B). As expected, a robust decrease in Nurr1 mRNA expression in the striatum and SN was found in the hemisphere exposed to MPTP as compared to saline-treated animals (*p* < 0.001) (Figure 5B). The administration of RA-NPs in MPTP-treated old mice significantly prevented the decrease of Nurr1 mRNA levels in the striatum as compared to MPTP (line set to 100%) (Figure 5C). In the same conditions, the RA released from NPs prevented, at least in part, the decrease of Nurr1 mRNA levels in the ipsilateral SN, but no statistical difference was observed when compared to MPTP.

## Discussion

In this study we have investigated the putative neuroprotective role of RA in a mouse model of PD taking advantage of a recently described nanoparticle delivery system developed by our group (Maia et al., 2011; Santos et al., 2012). RA regulates multiple biological processes including cell proliferation and differentiation, by virtue of its ability to modulate the rate of transcription of numerous target genes. It was also described that RA has a protective role against neurodegeneration of mDA neurons in the SN (Yin et al., 2012). However, in order to achieve protective or trophic effects, RA requires a well regulated narrow concentration window which posse difficulties in the delivery of therapeutic doses. Recently we developed a nanoparticle formulation that avoids the use of large concentrations of RA as well as controls the use of toxic solvents such as DMSO and ensures intracellular transport and controlled release of RA (Maia et al., 2011). We showed that this RA-NPs formulation enhances subventricular zone neurogenesis both *in vitro* and *in vivo*, without affecting cell survival and proliferation at the concentrations used in this study (Santos et al., 2012). However, there are no studies using this delivery system for DA neuroprotection in a context of PD. PD is mainly characterized by a progressive and preferential loss of DA neurons in SNpc which project their terminals to the striatum (Jankovic, 2008). In this sense, in the first part of this study, we investigated whether RA-NPs protect TH DA neurons from MPTP-induced lesion by performing TH immunohistostainings in midbrain and striatal sections. We demonstrated that intrastriatal injections with RA-NPs before MPTP administration significantly reduced the loss of TH+ cells and TH protein levels in the ipsilateral SN to levels similar to control. Moreover this neuroprotective effect induced by 100 ng/ml RA-NPs was more robust than the effect of 10 μM solubilized RA. In fact, the amount of RA payload present in 100 ng/ml of RA-NPs corresponds to 4 nM of RA and this concentration is 2500-fold lower than the concentration of 10 μM solubilized RA (Maia et al., 2011). Similarly, there was a significant reduction in intensity and area occupied by TH+ fibers in contralateral striatum of MPTP-treated mice and this reduction was counteracted by the presence of RA-NPs. Our results are in line with previous findings by others showing that RA can be an effective neuroprotective agent for DA neurons. In fact, a recent report showed that the intranasal delivery of 9cisRA, a less effective RA isomer, can also protect DA neurons against neurodegeneration induced by 6-OHDA (Yin et al., 2012). In the present study we used the atRA isomer, the main biologically active form, since it is more stable than 9cisRA isomer and can diffuse easily through the cellular membrane due to its lipophilic nature. Moreover, robust results were observed by performing a single intrastriatal administration of RA-NPs before MPTP-induced injury. This approach seems to be particularly feasible because numerous studies have demonstrated that intracerebral administration of several molecules (e.g., Neuropeptide Y, INI-0602) is able to prevent DA loss in the lesioned nigrostriatal DA system in PD animals models (Decressac et al., 2012; Yin et al., 2012; Suzuki et al., 2014). In fact, RA-NPs were injected in the striatum to maximize the amount of RA that could reach the dopaminergic terminals. Several authors using MPTP intoxication protocols reported that there is a higher degree of degeneration of DA terminals, concomitantly with the severe loss of striatal DA content, than of the TH cell bodies found at the SN. Therefore, dopaminergic degeneration is more severe at the level of nerve endings than at the level of the nerve perikarya, a concept compatible with the mechanism of MPTP action. Thus, the stereotaxic injection of RA-NPs in the striatum could enroll a neuroprotective effect directly in DA terminals in an early stage of DA degeneration. Moreover, as soon as reliable biomarkers for PD will be discovered, this RA-NPs formulation can be also used in an early stage of PD pathology to prevent or decelerate the progression of this disease. It has long been recognized that high concentrations or overexposure to RA causes widespread toxic effects in humans (Penniston and Tanumihardjo, 2006). Since the amount of RA per milligram of NPs is low, the use of this formulation can help to overcome these toxic effects. Additional delivery routes, such as the intranasal delivery, should be tested to clearly demonstrate the safety of our RA-NPs. However, the bioavailability of RA-NPs in the nigrostriatal pathway of PD patients that may present olfactory dysfunction, an early sign of PD, should be taken in consideration. This NPs formulation was also designed to facilitate the cellular internalization for delivery of RA within cells (Maia et al., 2011). We cannot assert that the beneficial effect provided by RA-NPs is due to a specific uptake by DA neurons, but it can create a beneficial environment to them, by modulating the expression of factors involved in survival of these neurons, namely glial cell line-derived neurotrophic factor (GDNF) and neurotrophin-3 (NT-3) or by suppressing inflammatory responses induced by activated glia (Thang et al., 2000; Xu and Drew, 2006). Moreover, DA neurons express RAR receptors, making these cells sensitive to the RA that can escape from the NPs to interact with these intranuclear receptors. In fact, Katsuki and colleagues (Katsuki et al., 2009) showed that both RARα and RARβ were highly expressed in DA neurons when compared with weak expression found in microglia and astrocytes. Also, treatment with Am80, a RARα/β agonist, triggered an increase of immunoreactivity against RARα and RARβ in the DA neurites, suggesting that RA released by nanoparticles that have been incorporated into DA terminals/neurites can activate RARs in DA neurons and trigger neuroprotection. This is in accordance with several authors who showed that RA signaling is involved in neurite outgrowth and axonal regeneration by regulating cAMP levels, AKT phosphorilation and expression of the inhibitory Nogo receptor (NgR) complex, among others (Puttagunta and Di Giovanni, 2011).

Unfortunately, till date the precise pathogenesis of PD remains largely unknown, however there are increasing evidences suggesting that dysfunction of some transcription factors may be involved in the differentiation and survival of mDA neurons which can be responsible for the development of PD (Jankovic et al., 2005; Luk et al., 2013). Amongst them, Nurr1 and Pitx3 are the most extensively studied ones. Nurr1 is one of the key regulators for the development and functional maintenance of DA neurons and is also considered as a crucial regulator for the expression of several genes involved in PD pathology including dopamine transporter and TH (Smits et al., 2003). This transcription factor was found predominantly expressed in DA neurons but can also be expressed by microglia cells (Zetterstrom et al., 1996; Saijo et al., 2009). Pitx3, another key factor for the development of DA neurons, is exclusively expressed in the DA cells present in the SN and ventral tegmental area of midbrain and is maintained throughout adult life in both rodents and humans (Smidt et al., 1997). This study is the first to show the modulation of Nurr1 and Pitx3 mRNA and protein expression using a RA-releasing NPs formulation. In a recent study, it was shown that the mRNA expression of both Nurr1 and Pitx3 were significantly decreased under PD conditions, suggesting that both genes were potential susceptibility genes for PD (Liu et al., 2012). Accordingly, in our experiments using both adult and old mice, we observed that the MPTP induced a robust decrease of both Nurr1 and Pitx3 mRNA levels, which is in line with other results previously reported (Gibrat et al., 2009; Rojas et al., 2012), and that the RA-NPs were able to counteract this decrease. This effect mediated by MPTP is more robust in the SN and striatum of older mice than in adult mice, which is in agreement with the fact that older mice are more susceptible to the toxic effects driven by MPTP as compared to adult mice. This may occur because monoamine oxidase-B (MAO-B) activity increases with age leading to the accumulation of high levels of MPP+ toxic form; thus both lethality and neurotoxicity of MPTP are age-dependent (Jarvis and Wagner, 1985). Because the risk of developing PD by itself is associated with advancing age, the finding that toxic actions of this neurotoxin also increase with age has generated considerable interest. So, it is important to evaluate the effects of RA-NPs in older animals since age is a risk factor of PD. For this reason, we also evaluated the effects of RA-NPs in Nurr1 and Pitx3 mRNA expression in MPTP-treated old mice. Surprisingly, the increased mRNA expression of Pitx3 was more robust in the SN of old mice treated with both RA-NPs and MPTP than in adult mice. Since the differences found between saline vs. MPTP-treated mice and MPTP- vs. MPTP + RA-NPs-treated mice in terms of number of TH+ cells and Pitx3 mRNA levels were correlated in the adult mice, we can predict that the same relationship may occur in old mice. Regarding Nurr1 expression, there was a drastic decrease in mRNA expression in MPTP-lesioned striatum of old mice compared with the Nurr1 mRNA expression in MPTP-lesioned striatum of adult mice. This may occur since the cells of old mice are more sensitive to the MPTP toxic effects than adults, and with the fact that MPTP also induces death of Nurr1-expressing microglia, thus resulting in a drastic decrease in Nurr1 mRNA expression in striatum (Serra et al., 2002). The pattern of Nurr1 mRNA expression is different in SN and striatum of adult and old mice treated with MPTP only and with RA-NPs. Our results suggest that Nurr1 and Pitx3 may be RA target genes and, therefore, RA can activate their transcription by binding to RAR-RXR heterodimers. The DA system is particularly susceptible to aging, possibly as a result of age-related decrease in Nurr1 expression that increases the vulnerability of the SN neurons to environmental stress (Chu et al., 2002). Nurr1 also has a role in inflammation by inhibiting the expression of pro-inflammatory neurotoxic mediators by microglia and astrocytes. Reduced Nurr1 expression results in exaggerated inflammatory responses in microglia that are further amplified by astrocytes leading to the production of factors that cause death of DA neurons (Saijo et al., 2009). Therefore, we can hypothesize that the decrease of Nurr1 with age can create a pro-inflammatory milieu responsible for a DA susceptibility to degeneration (Blasko et al., 2004). Several NPs formulations have been reported to release drugs in the brain as potential therapeutic strategies for PD (Garbayo et al., 2009; Regnier-Delplace et al., 2013). Concerns related to solubility, stability or bioavailability of some molecules make the use of biomaterials more frequent. However, the balance between biomaterials and payload molecule must be well established since the bioaccumulation and concomitant toxicity can generate undesired side-effects.

## Conclusions

**Figure 6:**
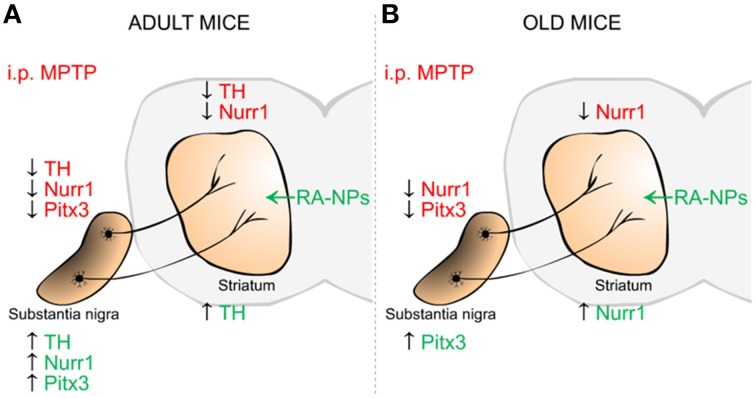
**Schematic summary of the effects mediated by RA-NPs against MPTP-induced injury in the dopaminergic (DA) nigrostriatal pathway of adult and old mice**. The i.p. injection of MPTP induced a decrease in TH DA neurons in SN and TH fibers in the striatum of adult mice (**A**-red color). This effect was accompanied by a decrease in mRNA expression of transcription factors involved in DA neuronal survival, Nurr1 and Pitx3, in SN and striatum of both age groups (**A,B**-red color). In the adult mice, the RA released from NPs protects the DA TH neurons in SN as well its neuronal TH fibers/axonal innervations in the striatum against MPTP-induced injury (**A**-green color). There was also an increase in Nurr1 and Pitx3 mRNA expression levels only in SN of adult mice (**A**-green color). Under the same conditions there was an increase in Pitx3 and Nurr1 mRNA expression in SN and striatum of old mice, respectively (**B**-green color).

Herein, we reported for the first time the neuroprotective effect on DA neurons triggered by RA released from NPs on a MPTP mouse model for PD. In addition, these RA-NPs also induced the expression of mRNA and protein of transcription factors responsible for DA neuronal specification and survival, namely Nurr1 and Pitx3 (Figure 6). Notably, this formulation offers a significant advantage over solubilized RA. Thus, RA-NPs could be an efficient/suitable strategy to prevent the onset of PD and potentially to open new perspectives for the treatment of other neurodegenerative diseases.

## Author contributions

ME: Conception and design; Collection and assembly of data; Data analysis and interpretation; Manuscript writing. AC: Collection and assembly of data; Data analysis and interpretation; Manuscript writing. TS and SR: Collection and assembly of data; Data analysis and interpretation. GB: Data analysis and interpretation; Critical reading of manuscript. LF: Provision of study material; Data analysis and interpretation; Administrative support; Critical reading of manuscript. LB: Conception and design; Provision of study material; Data analysis and interpretation; Financial support; Administrative support; Manuscript writing; Final approval of manuscript.

### Conflict of interest statement

The authors declare that the research was conducted in the absence of any commercial or financial relationships that could be construed as a potential conflict of interest.
